# Free DNA in Cystic Fibrosis Airway Fluids Correlates with Airflow Obstruction

**DOI:** 10.1155/2015/408935

**Published:** 2015-03-31

**Authors:** Veronica Marcos, Zhe Zhou-Suckow, Ali Önder Yildirim, Alexander Bohla, Andreas Hector, Ljubomir Vitkov, Wolf Dietrich Krautgartner, Walter Stoiber, Matthias Griese, Oliver Eickelberg, Marcus A. Mall, Dominik Hartl

**Affiliations:** ^1^Department of Pediatric Pulmonology, Hauner Children's Hospital, Ludwig Maximilians University, The German Center for Lung Research (DZL), 80377 Munich, Germany; ^2^Department of Translational Pulmonology, Translational Lung Research Center Heidelberg (TLRC), University of Heidelberg, The German Center for Lung Research (DZL), 69120 Heidelberg, Germany; ^3^Comprehensive Pneumology Center, Institute of Lung Biology and Disease (iLBD), University Hospital, Ludwig Maximilians University and Helmholtz Zentrum München, The German Center for Lung Research (DZL), 81377 Munich, Germany; ^4^Children's Hospital and Interdisciplinary Center for Infectious Diseases, University of Tübingen, 72076 Tübingen, Germany; ^5^Department of Operative Dentistry & Periodontology, Saarland University, 66424 Homburg, Germany; ^6^Biomedical Ultrastructure Research Lab, Division of Animal Structure and Function, Department of Cell Biology, University of Salzburg, 5020 Salzburg, Austria

## Abstract

Chronic obstructive lung disease determines morbidity and mortality of patients with cystic fibrosis (CF). CF airways are characterized by a nonresolving neutrophilic inflammation. After pathogen contact or prolonged activation, neutrophils release DNA fibres decorated with antimicrobial proteins, forming neutrophil extracellular traps (NETs). NETs have been described to act in a beneficial way for innate host defense by bactericidal, fungicidal, and virucidal actions. On the other hand, excessive NET formation has been linked to the pathogenesis of autoinflammatory and autoimmune disease conditions. We quantified free DNA structures characteristic of NETs in airway fluids of CF patients and a mouse model with CF-like lung disease. Free DNA levels correlated with airflow obstruction, fungal colonization, and CXC chemokine levels in CF patients and CF-like mice. When viewed in combination, our results demonstrate that neutrophilic inflammation in CF airways is associated with abundant free DNA characteristic for NETosis, and suggest that free DNA may be implicated in lung function decline in patients with CF.

## 1. Introduction

Cystic fibrosis (CF) is a fatal disorder characterized by chronic and progressive lung disease that determines morbidity and mortality of these patients [1]. Airways of CF patients show a chronic nonresolving neutrophilic inflammation, which increases upon infection and disease progression. Neutrophil products, such as elastase, chitinase-like proteins and chemokines, have been identified as important risk factors of lung damage and lung function decline and are suggested as biomarkers based on both cross-sectional and longitudinal studies in patients with CF [2–7] and mice with CF-like lung disease [8]. Previous studies also provided evidence that free extracellular DNA is highly increased in CF airway specimen [9], initially referred to as DNA derived from necrotic cells. However, several studies have now established that CF airway secretions contain meshwork structures reminiscent of NETs [10–14].

Neutrophils represent the first line of cellular host defense against bacteria and fungi. Traditionally, neutrophils have been known to combat pathogens intracellularly by phagocytosis, a paradigm that was extended and challenged by the finding that neutrophils can immobilize and kill pathogens extracellularly through NET formation (NETosis) [15, 16]. These released NETs consist of a nuclear DNA backbone equipped with characteristic granule and cytoplasmic proteins. While NETosis has been initially described as a novel form of cell death [17], recent studies demonstrated that also living neutrophils, eosinophils, and basophils can form extracellular traps (ETs) by expelling their mitochondrial DNA [18–23]. Viable/nonlytic rapid NET formation has been further found in response to* Staphylococcus aureus* infection, where phagocytosis, chemotaxis, and NET formation worked in a collaborative manner [24, 25].

In this study, we investigated CF airway inflammation with a focus on the abundance of free DNA structures characteristic for NETs in different airway specimen (sputum and BAL) obtained from patients with CF and *β*ENaC-transgenic (*β*ENaC-Tg) mice with CF-like lung disease [26–28] and correlated DNA levels with proinflammatory CXC chemokines, characteristic CF pathogens, and measurements of lung function. Our results demonstrate that free airway DNA levels correlate with obstructive lung disease and proinflammatory chemokines in CF patients and CF mice and could serve as therapeutic target and potential biomarker in CF lung disease.

## 2. Methods

### 2.1. NET Characterization

NETs were visualized and characterized by staining of extracellular DNA, citrullinated histones, myeloperoxidase, or elastase. The quantification of free DNA was performed using the Quant-iT PicoGreen assay (Molecular Probes, Inc., Eugene, OR, USA) based on a green fluorescent dye that binds DNA. For CLSM, samples were collected with poly-D-lysine-precoated cover slides placed on freshly harvested human sputum and were left in place for 5–10 min in order to adhere. The cover slides were washed in PBS at pH 7.4 and transferred into a fixative of 4% paraformaldehyde for 2 hours. The fixed samples were washed with PBS, permeabilized (0.5% Triton X-100 in PBS), and blocked (10% normal goat serum, 10 mM glycine in diluent containing 0.5% bovine serum albumin, 0.5% normal goat serum, and 0.5% Triton X-100 in PBS). DNA was stained with DAPI (Sigma-Aldrich, Vienna, Austria). For visualisation of citrullinated histones, the samples were incubated with the rabbit anti-human citrullinated histone H3 antibody (ab77164, Abcam, Cambridge, UK). This antibody was detected in CLSM by means of a secondary anti-rabbit FITC antibody (ab6717, Abcam, Cambridge, UK). For visualisation of neutrophil elastase or myeloperoxidase, the samples were incubated with rabbit antineutrophil elastase (ab21595, Abcam, Cambridge, UK) or mouse antimyeloperoxidase (ab25989, Abcam, Cambridge, UK) antibodies. Negative controls were initially incubated in 500 U/mL DNase (DNase I recombinant, grade I, Roche Diagnostics GmbH, Vienna, Austria) for 20 min at room temperature (RT) and then stopped with 50 mM EDTA in excess and thereafter treated as mentioned above. The specimens were analysed with a CLSM (Zeiss LSM 510 meta UV, Carl Zeiss GmbH, Vienna, Austria). Cross talk between different channels was avoided by using multitracking modus. Relative fluorescence was quantified using Zeiss LSM software application. For scanning electron microscopy (SEM) studies, sputum samples, collected as described above, were fixed for two hours with Karnovsky fixative. The fixed samples were washed with 0.1 M sodium cacodylate at pH7.6 and blocked in 1% BSA for 20 min at RT. Then, samples were dehydrated in ascending series of ethyl alcohol, critical-point-dried, and subsequently sputtered with gold. The specimens were examined in a scanning electron microscope ESEM XL30 (FEI Company, PHILIPS, Eindhoven, Netherlands) operating at 20 kV. The negative controls were digested with DNase and thereafter processed in the same way. For transmission electron microscopy (TEM) studies, sputum samples were collected with formvar-coated grids placed on freshly harvested sputum and left in place for 60 sec in order to adhere. The grids were then immediately transferred into a fixative of 4% paraformaldehyde in PBS at pH 7.4 for two hours. The fixed samples were washed with PBS, permeabilized, and blocked (10% normal goat serum, 10 mM glycine, 0.2% Tween 20 in diluent containing 0.5% bovine serum albumin, and 0.5% Triton X-100 in PBS). For visualisation of citrullinated histone H3, the grids were incubated with rabbit anti-human citrullinated histone H3 (citrulline 2 + 8 + 17) antibody [CitH3] (ab77164, Abcam, Cambridge, UK) and a gold-conjugated secondary antibody (ab27237, Abcam, Cambridge, UK, gold sphere diameter 20 nm or ab27235, Abcam, Cambridge, UK, for 5 nm gold sphere diameter). Finally, the grids were stained with 1% uranyl acetate (Sigma-Aldrich, Vienna, Austria). The negative controls were digested with DNase and thereafter processed in the same way. A second type of negative controls was obtained by omitting the primary antibody. For ultrathin sputum sections, sputum samples were stained with the ruthenium-red-osmium-tetroxide technique to enable the visualization of NETs and bacterial glycocalyx. Briefly, the samples were fixed with 1.2% glutaraldehyde (buffered at pH 6.5 with 0.1 M sodium cacodylate) with the addition of 0.05% ruthenium-red for 2 hours at RT. Postfixation was performed with 1% osmium-tetroxide (buffered at pH 6.5 with 0.1 M sodium cacodylate) and 0.05% ruthenium-red for 2 h at RT. The specimens were routinely embedded in Epon 812. Ultrathin sections were examined with a transmission electron microscope LEO EM 910 (LEO Elektronenmikroskopie Ltd., Oberkochen, Germany).

### 2.2. Quantification of CXCR2 Ligands

Levels of human or murine CXCR2 ligands were quantified by commercial sandwich ELISA kits according to the manufacturer's instructions as previously described [29].

### 2.3. Human Subjects

CF patients and healthy control subjects were included in the study (Table 1). The diagnosis of CF was based on typical clinical symptoms and positive sweat tests or disease-causing mutations in the* CFTR* gene. Inclusion criteria for CF patients were stable concomitant therapy at least two weeks prior to the study and a forced expiratory volume in 1 second (FEV_1_) > 25% of predicted value. Ten control subjects without pulmonary diseases were selected as the control group. These subjects had no pulmonary disease and were free of respiratory tract infections. Chronic bacterial and fungal colonization were diagnosed using the Leeds criteria [30], if the organism was present in more than 50% of patient samples in the year prior to analysis. Bacterial and fungal species were analyzed using culture-based methods. The study was approved by the Institutional Review Board and by the Ethics Committees of the Medical Faculty, Ludwig-Maximilians University, Munich, and the University of Tübingen, Germany. Written informed consent was obtained from all patients and control subjects prior to the study. This study was conducted in accordance with the amended Declaration of Helsinki.

### 2.4. CF Airway Specimen

Induced sputum was obtained after inhalation of 5.85% hypertonic sodium chloride for 15 min. Low-speed (4°C, 500 g for 10 min) supernatants obtained from induced sputum were further centrifuged at 4°C, 4000 g for 20 min. Cell-free sputum supernatant was stored at −80°C until analysis. Bronchoscopy and BAL (4 × 1 mL of 0.9% NaCl per kg body weight) were performed as described previously [31, 32]. Because of the high percentage of neutrophils the first fraction of BAL was used for subsequent analyses.

### 2.5. Experimental Animals

All animal studies were approved by the Regierungspräsidium Karlsruhe or Munich, Germany. The generation of *β*ENaC-Tg mice (line 6608) has been previously described [26]. The colony was maintained on a mixed genetic background (C3H/HeN × C57BL/6N), and *β*ENaC-Tg mice were identified by PCR. Wild-type littermates served as controls in all experiments. Mice were housed in a pathogen-free animal facility and had free access to chow and water.

### 2.6. Bronchoalveolar Lavage and Cytokine Measurements

For bronchoalveolar lavage (BAL), mice were deeply anesthetized via intraperitoneal injection of a combination of ketamine/xylazine (120 mg/kg and 16 mg/kg, resp.), the trachea was cannulated, and lungs were carefully lavaged twice with 800 *μ*L PBS. KC and MIP2 concentrations were measured in BAL supernatant using ELISA according to manufacturer's instructions and total cell counts were determined and differential cell counts performed on cytospin preparations. Studies were performed by investigators who were blinded with respect to the genotype and the treatment of the mice.

### 2.7. Pulmonary Function Studies in Mice

We used invasive pulmonary function devices (Forced Maneuver System, Buxco Research Systems, Wilmington, NC). Mouse pulmonary function testing was performed and analyzed as published previously [33]. All mice were anesthetized with i.p. MMF (medetomidine, midazolam, and fentanyl), intubated, and placed in a forced pulmonary maneuver system. In a heated plethysmograph chamber, mice were ventilated at an average rate of 140 breaths per minute, and flow, mouth, esophageal pressure and heart rate were monitored to measure forced expiratory volume at 100 ms (FEV100).

### 2.8. Statistical Analysis

Comparisons among all groups were performed with ANOVA and comparisons between two patient groups were performed with the two-sided *t*-test. Correlation analysis was performed by calculating the two-tailed Pearson correlation coefficient. Statistical analysis was performed with Prism 4.0 (GraphPad Software) and STATA version 8.2 for Windows (STATA Corporation).

## 3. Results

**Figure 1:**
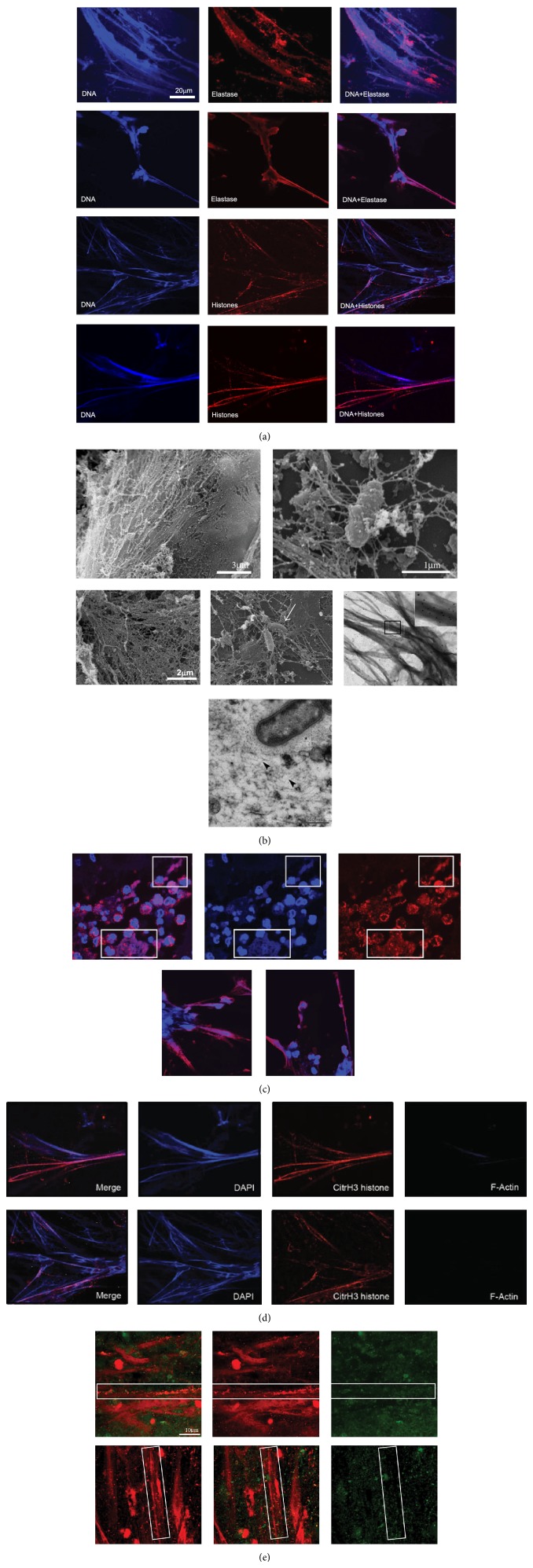
Free NET-like DNA structures in CF lung disease. (a) Immunological characterization of free DNA structures in CF airway fluids by CLSM. Upper two panel rows: NET-like DNA structures in induced CF sputum. Blue: DAPI stains DNA-NET backbone. Red: elastase. Lower two panel rows: Blue: DAPI, red: citrullinated histones. Scale bar: 20 *μ*m. (b) Ultrastructure of free NET-like DNA structures. Upper and middle panels: SEM images of CF airway fluids. Arrow marks bacteria entrapped in DNA-NET-like structures. Scale bar: 2 *μ*m; Middle right panel: TEM staining of citrullinated histones in CF airway fluids (sputa). Lower panel: Ultrathin sections of CF airway DNA-NET-like structures. The NETs and the bacterial extracellular polysaccharides are visualized by the ruthenium-red-osmium-tetroxide technique. Bacteria embedded in a dense wickerwork of NETs. Arrowheads mark NETs; asterisk: bacterial extracellular polysaccharide. (c)* Upper panel:* free DNA structures in CF lung tissue. Red: MPO (as characteristic NET component). Blue: DAPI (DNA). Inlays mark characteristic NET-areas.* Lower panel:* CF airway NETs in CF BAL fluids. Red: elastase (as characteristic NET component). Blue: DAPI (DNA). (d) Costainings of DAPI, citrullinated histones, and F-actin. Scale bar: 20 *μ*m. (e) Dead/live staining of CF airway fluids (induced sputum). Free DNA and dead bacteria appear red; vital bacteria appear green.

Increased extracellular DNA fibres with morphological NET characteristics were found in airway specimen from CF patients (Figures 1(a) and 1(b)). Positive costaining for neutrophil elastase (Figure 1(a)) and citrullinated histones (Figures 1(a), 1(b), and 1(d)), as characteristic NET markers, and negative staining for F-actin (Figure 1(d)) supported the notion that the CF airway DNA fibres represented NETs rather than necrosis-derived DNA. Treatment of CF airway fluids with DNase dissolved these DNA-NET-like structures further confirming their nature as DNA strands (*data not shown*). Ultrastructural imaging methods supported these findings and revealed a complex meshwork of DNA strands with bacteria entangled (Figure 1(b)). Free DNA was present in different CF airway compartments, in particular in sputum (Figure 1(a)), bronchoalveolar lavage fluid (BAL) (Figure 1(c),* lower panel*), and lung tissue (Figure 1(c) *upper panel*). Remarkably, most abundant free DNA was found in CF sputa, whereas in BAL and lung tissue, lower amounts of free DNA strands were detected, which is consistent with the observation that neutrophilic inflammation is most prominent in the proximal/bronchial airway compartments in CF lung disease. In BAL fluid, we observed lower amounts of DNA NET-like structures, but observed that elastase was associated to free DNA structures and also appeared to colocalize with the neutrophil's cellular membrane (Figure 1(c),* lower panel*). In CF airway fluids, we found DNA/NET-like structures associated with bacteria (Figure 1(e)). However, associations of free DNA structures with both live and dead bacteria were noted (Figure 1(e)).

**Figure 2:**
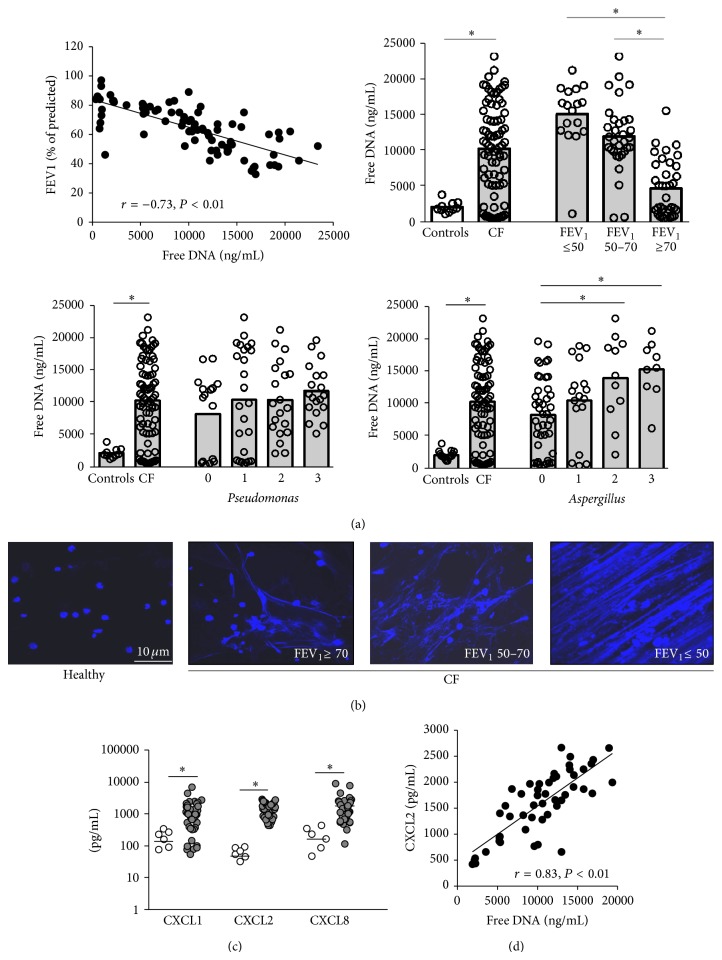
Free DNA, disease severity, and infection in CF lung disease. (a) Stratification of CF patients (*n* = 80). Upper left: correlation of free DNA levels with FEV_1_ in sputum supernatants from CF patients. Upper right: free DNA levels in sputum supernatants from healthy controls and CF patients, stratified by FEV_1_. Lower panels: free DNA levels in sputum supernatants from healthy controls and CF patients, stratified by* Pseudomonas aeruginosa* or* Aspergillus fumigatus* infection/colonization status (*Leeds* criteria, 0: never, 1: negative, 2: intermittent, and 3: chronic). (b) Representative CLSM images of airway fluids from one healthy individual and three different CF patients, stratified for lung function, are depicted. Scale bar: 10 *μ*m; FEV_1_: forced expiratory volume in 1 second (% of prediction). DAPI staining of nuclei and extracellular DNA strands in CF sputa. (c) Chemokine levels in sputum supernatants from healthy controls (white, *n* = 6) or CF patients (grey, *n* = 50). (d) Correlation of free DNA with CXCL2 levels in airway fluids (cell-free sputum supernatants) from CF patients (*n* = 50). ^∗^ *P* < 0.05.

Stratifying CF patients for disease severity, we found that CF patients with poor pulmonary function had higher levels of free DNA in their airway fluids than patients with mild lung disease (Figure 2(a)). We further found that the airway free DNA levels were associated with fungal colonization with* Aspergillus fumigatus* but surprisingly not with bacterial infection (Figure 2(a)). Representative NET-DNA (DAPI) staining of CF patient groups stratified for lung disease severity is shown in Figure 2(b). Highly increased levels of the CXC chemokines CXCL1 (GRO-alpha), CXCL2 (GRO-beta), and CXCL8 (IL-8) were detected in CF airway fluids (Figure 2(c)). Since CXCR1 is proteolytically cleaved on CF airway neutrophils [4, 32], CXCR2 remains the main binding site for these chemokines in the CF airway microenvironment. CF airway NETs correlated positively with levels of the proinflammatory chemokine CXCL2 (Figure 2(d)). These findings provide evidence that CF lung disease features free airway DNA levels characteristic for NETosis and suggest that increased free DNA levels are associated with poor lung function of CF patients.

**Figure 3:**
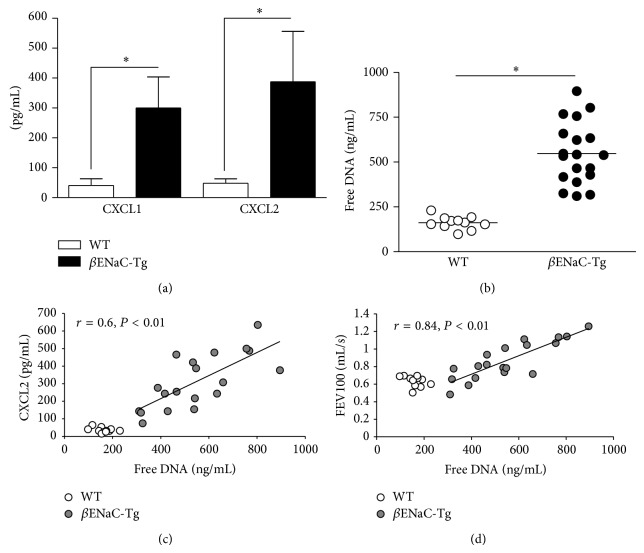
Free DNA in murine CF lung disease* in vivo*. (a) CXCR2 chemokines in BALF from *β*ENaC-transgenic (*β*ENaC-Tg, *n* = 19) and wild-type (WT, *n* = 11) mice. Levels of CXCL1 (KC) and CXCL2 (MIP-2) were quantified by ELISA. (b) Free DNA in BALs from *β*ENaC-Tg mice. Free DNA was quantified in BALF from *β*ENaC-Tg (filled circles, *n* = 19) and wild-type (empty circles, *n* = 11) mice. (c) Correlation of free DNA with CXCL2 levels in BAL. (d) Correlation of free DNA with lung function (FEV100). ^∗^ *P* < 0.05.

To investigate the role of free DNA/NET formation* in vivo*, we used transgenic mice with airway-specific overexpression of the amiloride-sensitive epithelial Na^+^ channel (*β*ENaC-Tg) as a model of CF-like lung disease [26, 27]. These mice phenocopy airway surface liquid depletion, mucociliary dysfunction, and chronic airway disease with neutrophilic airway inflammation, mucus obstruction, and structural lung damage [26, 27, 34]. Similar to human CF airway fluids, levels of the CXCR2 ligands CXCL1 and CXCL2 (Figure 3(a)) and free DNA (Figure 3(b)) were highly increased in the BAL airway fluids of *β*ENaC-Tg mice compared to wild-type controls. Also consistent with the data obtained from human CF lung disease samples, the extent of free DNA in murine CF airway fluids correlated positively with the levels of the CXCR2 ligand CXCL2 (Figure 3(c)) and with pulmonary obstruction parameters (FEV100) (Figure 3(d)), whereas no correlation was found between free DNA and levels of CXCL1 or parameters of pulmonary restriction parameters (data not shown).

## 4. Discussion

NETosis is regarded as a double-edged sword in human disease: on one hand, NETs can capture, immobilize, and kill pathogens; on the other hand, uncontrolled and infection-independent NET formation has the potential to harm host tissue through histones [35], proteases, or other mechanisms [21, 36, 37]. Related to the latter mechanism, NET formation has recently been implicated in the pathogenesis of autoinflammatory and autoimmune disease conditions, such as lupus, preeclampsia, septic shock, and autoimmune vasculitis [36–40]. Beyond these conditions, NETs were found, in concert with platelets and monocytes, to play a role in thrombus formation and deep vein thrombosis [41, 42]. However, antihost defense and autoinflammatory conditions are not dichotomous in their nature and temporarily overlap upon resolution of infection or progression into chronic infection, especially in immunocompromised conditions. Viewing these findings in combination, the balance between targeted antimicrobial host defense and nontargeted tissue damage of NET is delicate, but it is essential for the understanding and therapeutic potential of NET formation in human disease conditions.

The potential role of NETs in inflammatory lung diseases has just begun to evolve [40]. Several studies have now provided evidence for NETosis or NETosis-like structures in CF lung disease and broadened the potential role of NET formation in the complex pathogenesis of infective CF lung disease [43]. We and others have demonstrated previously that free DNA NET-like structures are abundantly detectable in CF airway secretions [10–14]. Recently, a further study found that CF sputum showed NETosis characteristics and implicated macrophage migration-inhibitory factor (MIF) in the formation of NETs in the context of CF lung disease [12]. Here, we confirm and extend these previous observations by showing that CF airway free DNA levels correlate with pulmonary obstruction in CF patients and mice. These observations are reasonable, given the continuous and nonresolving neutrophil recruitment and activation within CF airways, being supported by the beneficial effect of recombinant inhaled DNase (Dornase alpha) in CF patients with the strongest evidence in moderate and severe disease severity [44–46]. Based on the increasing extent of neutrophilic inflammation and pulmonary obstruction, the evidence of free NET-like DNA structures in CF airways tempts us to speculate that in earlier and milder stages of CF lung disease, NET formation may act beneficial in providing extracellular antibacterial and antifungal host defense. At this time, DNase may be used with more caution, since encaptured pathogens might be freed and could cause additional host damage. On the other hand, in later moderate to severe stages of CF lung disease, the amount of mucus and DNA accumulation causes airway obstruction, rendering DNase efficient in cleaving DNA traps, and this effect probably overweighs the antimicrobial actions of NET formation, a hypothesis that awaits to be tested in further* in vivo* studies and clinical trials. Our correlations between free DNA levels and pulmonary obstruction parameters in human patients with CF and a mouse model of CF lung disease suggest that, at least in our CF cohort with a more advanced lung disease severity, NETs may cause more harm than good to the host. The obvious limitations of our study and the whole approach of free DNA analysis in CF airway fluids remain that the free extracellular DNA could result from different forms of cell death in addition to NETosis, including necrosis, pyroptosis, and others. In addition, our studies image NET formation using fixation of biological samples and are thereby limited by the fact that DNA structures reminiscent of NETosis cannot be clearly attributed to active NET formation, since this would require live cell imaging approaches. Nevertheless, several other previous publications have provided indirect evidence indicating the presence of NET structures in CF airway fluids [10–14], which is supported methodologically by our studies using confocal laser scanning, scanning electron microscopy, transmission electron microscopy, and atomic force microscopy, decreasing the probability of fixation/staining artefacts.

As CF lung disease is typically associated with chronic colonizations and infections with* Pseudomonas aeruginosa*, the effect of this pathogen on NET formation has been investigated using different methodologies and modelling systems [11, 13, 47–49]. The latter studies found that* Pseudomonas aeruginosa* efficiently induced NET formation, particularly in solution* in vitro*, and demonstrated that NETs can kill this pathogen through an extracellular DNA-mediated mechanism. At single cell level, authors also showed that neutrophils from CF patients had no cell intrinsic alteration in NET formation [11], supporting the notion that the NET accumulation found in CF airway fluids is not specific to CFTR mutation-based disease conditions, but rather represent a prototypical picture of severe and chronic neutrophilic inflammation in a body compartment. Serum DNases and/or antioxidants have been shown to dampen/inhibit NET formation. As CF airway fluids show a low antioxidant activity and are substantially different in their biochemical properties, we speculate that this particular microenvironment favors NET formation. While we did not find a statistically significant association of* Pseudomonas aeruginosa* infection status with free DNA levels, a positive correlation was found for fungal colonization with* Aspergillus fumigatus*, which is in line with a recent study showing that NETs are mainly formed in response to large pathogens, such as fungi [50].

A recent study analyzing neutrophils from a patient with Papillon-Lefevre syndrome (PLS), lacking serine proteases, showed that neutrophils from this patient failed to produce NETs [51], corroborating the concept that serine proteases, particularly elastase, regulate NET formation [50, 52–54]. Papayannopoulos and coworkers demonstrated that neutrophil elastase, which is highly increased in CF airway fluids and represents a promising therapeutic target [1, 4], enhances sputum solubilization by cleaving histones to enhance the access of exogenous nucleases to DNA [10]. The researchers also showed that neutrophil elastase is bound to DNA, which downregulates its proteolytic activity and could restrict host tissue damage. This study has been extended by a recent report demonstrating that NETs represent a reservoir of active proteases and DNase treatment increases free proteolytic activities [55], suggesting that CF patients under DNase treatment could benefit from the addition of protease inhibitors [56].

Previous studies found a correlation between airway DNA levels, neutrophilic inflammation, and lung function parameters in CF patients [9, 57, 58]. Our study confirms and extends these findings by showing a correlation between free DNA levels with lung function, chemokine levels, and fungal colonization in CF patients. The correlation between the neutrophil chemokine CXCL2 and free DNA levels in CF airway fluids could, on one hand, reflect increased CXCL2-mediated neutrophil chemotaxis or could also, on the other hand, involve CXCL2 itself as potential trigger for DNA release in neutrophils. As there is a lack of convincing data supporting the latter hypothesis, the underlying mechanistic basis for this correlation remains to be dissected in the future.

Besides NET-associated products, such as proteases, that can cause harm to the host, a recent study identified surfactant protein D as key protein binding to NETs, thereby promoting NET-mediated trapping of* P. aeruginosa* [59]. This has ample implications for CF lung disease, since surfactant protein D has been found to be degraded/cleaved by both serine and matrix metalloproteases in CF airway fluids [60–64], which, as a result, could impair the antibacterial NETosis-related effects of surfactant protein D in CF airways* in vivo*. Regarding surfactant protein D and beyond, proteins that may interfere with NET formation and/or NET activities could be attractive therapeutic targets for advanced CF lung disease.

Our view of NET formation has been further extended by the description of rapid NET formation* in vitro* [65] and* in vivo* [24, 25], unraveling a so far unappreciated mode of collaboration between NET formation, chemotaxis, and phagocytosis. While there is convincing data in skin infection models in mice, evidence on human neutrophils is still limited. The potential role of rapid NET formation for CF lung disease remains to be dissected.

In summary, our study, in line with previous investigations, demonstrates that free DNA with NET-like characteristics represents an extracellular component of CF airway fluids. In advanced stages of CF lung disease, NETs seem to do more harm than good, but experimental data for a causative relationship is lacking. Approaches to interfere with NET formation or NET-associated products, such as DNases, antiproteases, supplementing surfactant protein D, targeting histones [66], or a combination thereof, could represent promising therapeutic strategies for CF lung disease and other chronic lung diseases associated with sustained neutrophilic inflammation [38, 40, 43].

## Figures and Tables

**Table 1:** Patient characteristics.

	Cystic fibrosis	Healthy controls
Number total	80	10
Age (years)	22 ± 10	28 ± 8
Gender (M : F)	48/32	6/4
FEV_1_ (% pred)	64.6 ± 15	n.d.
FVC (% of pred)	73 ± 20	n.d.
MEF_25–75_ (% pred)	33 ± 12	n.d.
Neutrophils (%) in sputum	88 ± 29	20 ± 9
dF508 *homozygous/heterozygous/other *	38/27/15	n.d.

Statistical analysis was performed with ANOVA and the two-sided *t-*test. M: male, F: female; FEV_1_: forced expiratory volume in 1 second (% of predicted); results are expressed as means ± standard deviation; n.d. not determined.
